# Prognostic and Immunological Roles of MMP-9 in Pan-Cancer

**DOI:** 10.1155/2022/2592962

**Published:** 2022-02-07

**Authors:** Yudan Zeng, Mengqian Gao, Dongtao Lin, Guoxia Du, Yongming Cai

**Affiliations:** ^1^School of Public Health, Guangdong Pharmaceutical University, Guangzhou, China; ^2^College of Medical Information Engineering, Guangdong Pharmaceutical University, Guangzhou, China; ^3^Guangdong Provincial TCM Precision Medicine Big Data Engineering Technology Research Center, Guangzhou, China; ^4^Key Specialty of Clinical Pharmacy, The First Affiliated Hospital of Guangdong Pharmaceutical University, China

## Abstract

**Background:**

Matrix metalloproteinase-9 (MMP-9) can degrade the extracellular matrix and participate in tumor progression. The relationship between MMP-9 and immune cells has been reported in various malignant tumors. However, there is a lack of comprehensive pan-cancer studies on the relationship between MMP-9 and cancer prognosis and immune infiltration.

**Method:**

We used data from TCGA and GTEx databases to comprehensively analyze the differential expression of MMP-9 in normal and cancerous tissues. Survival analysis was performed to understand the prognostic role of MMP-9 in different tumors. We then analyzed the expression of MMP-9 across different tumors and at different clinical stages. Based on the results, we assessed the correlation between MMP-9 expression and immune-associated genes and immunocytes. Finally, we calculated the tumor mutation burden (TMB) of 33 cancer types and analyzed the correlation between MMP-9 and TMB, DNA microsatellite instability, and DNA repair genes.

**Results:**

MMP-9 significantly affected the prognosis and metastasis of various cancers. It was associated based on overall survival, disease-specific survival in five tumors, progression-free interval in seven tumors, and clinical stage in eight tumors, as well as with prognosis and metastasis in adrenocortical carcinoma and kidney renal clear cell carcinoma. It was also coexpressed with immune-related genes and DNA repair genes. The expression of MMP-9 was positively correlated with the markers of T cells, tumor-associated macrophages, Th1 cells, and T cell exhaustion. Furthermore, MMP-9 expression was highly correlated with macrophage M0 in 28 tumors. In addition, its expression was associated with TMB in eight cancer types and DNA microsatellite instability in six cancer types.

**Conclusion:**

MMP-9 is related to immune infiltration in pan-cancer and can be used as a biomarker related to cancer prognosis and metastasis. Our findings provide prognostic molecular markers and new ideas for immunotherapy.

## 1. Introduction

Matrix metalloproteinase-9 (MMP-9) [1] is a significant matrix metalloproteinase that is involved in many biological processes by degrading the extracellular matrix. MMP-9 plays an important role in the onset, progression, and metastasis of gastric [2], lung [3], colon [4], and breast cancers [5]. Metastasis is a major cause of mortality in patients with cancer. MMP-9 promotes metastasis and angiogenesis through decomposition of the extracellular matrix [6, 7]. Infiltration of immune cells can also affect cancer metastasis and prognosis. Recently, many studies [8–10] evaluated the potential of MMP-9 as a biomarker for the prognosis of various cancers, including cervical [11, 12], ovarian [13, 14], pancreatic [15], and breast cancers [16].

In recent years, the incidence of cancer and its morbidity and mortality have shown an increasing trend. Cancer is a major cause of death worldwide and is second only to cardiovascular disease. The WHO estimates that malignant tumors will become the main cause of global mortality after 2030 [17]. The tumor microenvironment (TME) influences tumor growth and development. Tumor-associated macrophages (TAMs) are macrophages that infiltrate the tumor tissue and most immune cells in the TME. Tekin et al. [18] found that macrophages release MMP-9 in pancreatic cancer. TAMs can support the proliferation, invasion, and metastasis of tumor cells. Therefore, the development of antitumor drugs that can target macrophage polarization is urgently required. Immunotherapy is highly suitable for patients with cancer because of its excellent efficacy. However, not all patients can benefit from immunotherapy and research has shown that tumor mutation burden (TMB) and DNA microsatellite instability (MSI) can be used as predictive markers for immunotherapy efficacy. TMB [19] has a good predictive value for immunotherapy in a variety of tumors. In addition, MSI [20] has been regarded as an important molecular marker for the prognosis and adjuvant treatment of colorectal cancer and other solid tumors. In view of the complexity of tumor progression, pan-cancer analysis has been widely used in cancer research and considerable progress has been made in understanding various tumor features, including cancer susceptibility variation, oncogenic pathway cooccurrence and mutual exclusion, and biological regulation network disorder [21–23].

**Figure 1:**
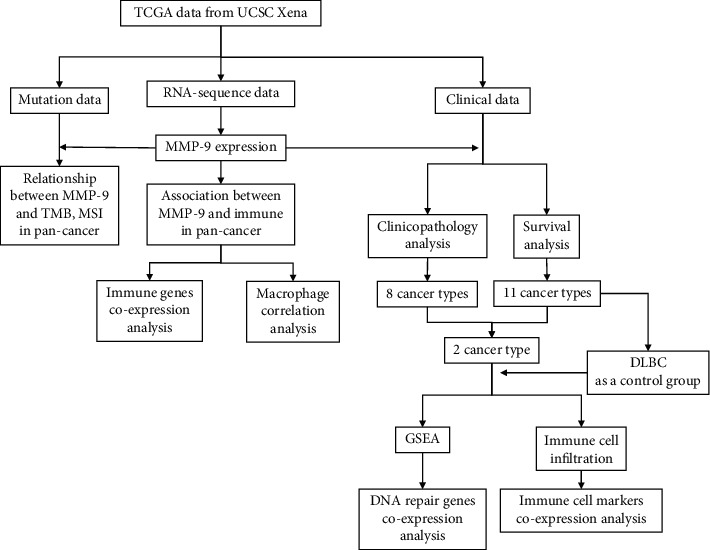
Flow chart of this article.

MMP-9 has been found to be closely related to immunity and tumor progression; however, most studies have focused on single cancers. Here, we systematically studied MMP-9 expression and its correlation with prognosis and metastasis in 33 cancer types to help us fully understand the role of MMP-9 in tumors. A flowchart of the study is shown in Figure 1. We also analyzed the relationship between MMP-9 expression and immune cell infiltration.

## 2. Materials and Methods

### 2.1. Data Acquisition

Gene expression profiles, mutation data, and clinical information of 33 cancers in TCGA database were downloaded from UCSC Xena [24] (http://xena.ucsc.edu/).The disease-specific survival (DSS) and progression-free interval (PFI) data were downloaded from TCGA Pan-Cancer (PANCAN) of UCSC Xena. After excluding cases with missing survival time data, 11,057 samples were included in the study.

### 2.2. Gene Expression Analysis

We used “wilcox.test” to analyze the differential expression of MMP-9 in normal and tumor tissue samples, as well as the differential expression of MMP-9 in different cancer types in TCGA database, and drew a box diagram.

In view of the small number of normal tissue samples in TCGA database, we included data from the GTEx (genotype-tissue expression) database [25] using the “Match TCGA normal and GTEx data” option in the GEPIA2 database [26] (http://gepia2.cancer-pku.cn/#analysis) for the differential analysis to ensure more reliable results.

### 2.3. Survival and Clinical Analysis

The expression of MMP-9 was extracted from the gene expression profile data, and the samples were divided into high- and low-expression groups according to the median MMP-9 expression. We used the Kaplan-Meier method to analyze the survival information and “survival” [27] and “survminer” to draw the survival curve. We also performed COX analysis of the survival data, and the R package “forestplot” was used to visualize the results.

A boxplot using tumor stage as a variable was graphed to observe the differences in MMP-9 expression at different clinical stages and analyze the relationship between the expression level of MMP-9 and tumor metastasis in different cancers. This was carried out using the R package “limma” [28].

### 2.4. Immunological Correlation Analysis

We used the “Gene” module of TIMER [29] (https://cistrome.shinyapps.io/timer/) to explore the correlation between MMP-9 expression and abundance of immune infiltrates in adrenocortical carcinoma (ACC), kidney renal clear cell carcinoma (KIRC), and lymphoid neoplasm diffuse large B-cell lymphoma (DLBC). In addition, we employed the “Immune-Gene” module in the TIMER2.0 database [30](http://timer.comp-genomics.org/) to explore the association between MMP-9 expression and macrophage immune infiltration.

The R package “CIBERSORT” [31] was used to evaluate the infiltration of immune cells in all samples. Coexpression analysis of MMP-9 and immune cells was performed using Spearman's correlation. In addition, we calculated the correlation coefficient between various immune markers and MMP-9 using “limma.”

### 2.5. Mutation Analysis

TMB refers to the number of somatic mutations that occur after germline mutations are removed from the tumor genome. We used PERL scripts to calculate the TMB of each sample. The MSI values were derived from TCGA database. We then analyzed the correlation between MMP-9 and TMB and MSI and designed a radar map using the R package “fmsb.”

### 2.6. Gene Set Enrichment Analysis (GSEA)

We used GSEA to group and classify the genes according to multiple functional gene sets, such as the GO gene set. We used the package “clusterProfiler” [32] of R (ver. 3.6.3) to analyze the GO enrichment of MMP-9 in ACC, KIRC, and DLBC.

### 2.7. Other Analyses

We extracted the expression of common immune checkpoint genes and DNA repair genes of 33 tumors and used Spearman correlation coefficients to evaluate their correlation with MMP-9 expression.

## 3. Results

### 3.1. mRNA Expression Levels of MMP-9 in Different Types of Human Cancers

**Figure 2:**
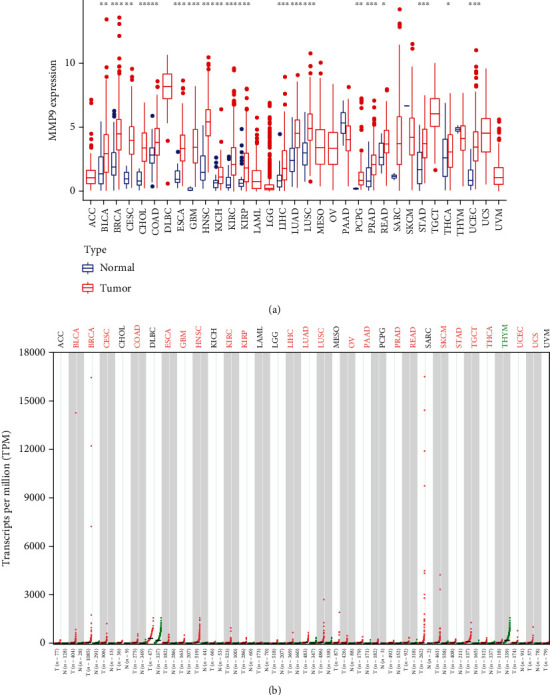
MMP-9 expression levels in different tumor types in various databases. (a) Expression level of MMP-9 in different tumors of TCGA database; MMP-9 expression in tumor samples is significantly higher than normal in many cancer types. The *P* values are indicated as ^∗^*P* < 0.05, ^∗∗^*P* < 0.01, and ^∗∗∗^*P* < 0.001. (b) Expression level of MMP-9 in different tumors of data matching TCGA normal and GTEx data by GEPIA2 database; marked red cancer means that MMP-9 is highly expressed in tumor tissues and marked green cancer represents that MMP-9 is highly expressed in normal tissues.

To determine the differences in the expression levels of MMP-9 in various human cancers, we examined the MMP-9 expression levels using the RNA-seq data of multiple malignancies from TCGA database. The differential expression of MMP-9 between tumor and adjacent normal tissues across tumor types is shown in Figure 2(a). Except for tumors without normal tissue data, MMP-9 expression was significantly higher in tumor samples than in normal samples.

Owing to the insufficiency of normal tissue data in TCGA database, we included data from the GTEx database to supplement TCGA data for the differential analysis (Figure 2(b)). MMP-9 was highly expressed in the tissues of bladder urothelial carcinoma (BLCA), breast invasive carcinoma (BRCA), cervical squamous cell carcinoma and endocervical adenocarcinoma (CESC), colon adenocarcinoma (COAD), esophageal carcinoma (ESCA), glioblastoma multiforme (GBM), head and neck squamous cell carcinoma (HNSC), KIRC, kidney renal papillary cell carcinoma (KIRP), liver hepatocellular carcinoma (LIHC), lung adenocarcinoma (LUAD), lung squamous cell carcinoma (LUSC), ovarian serous cystadenocarcinoma (OV), pancreatic adenocarcinoma, rectum adenocarcinoma, skin cutaneous melanoma (SKCM), stomach adenocarcinoma, testicular germ cell tumors, uterine corpus endometrial carcinoma (UCEC), and uterine carcinoma compared with normal tissues. Interestingly, the expression of MMP-9 was higher in the normal tissues of thymoma than in tumor tissues.

### 3.2. Association between MMP-9 Expression and Cancer Prognosis

**Figure 3:**
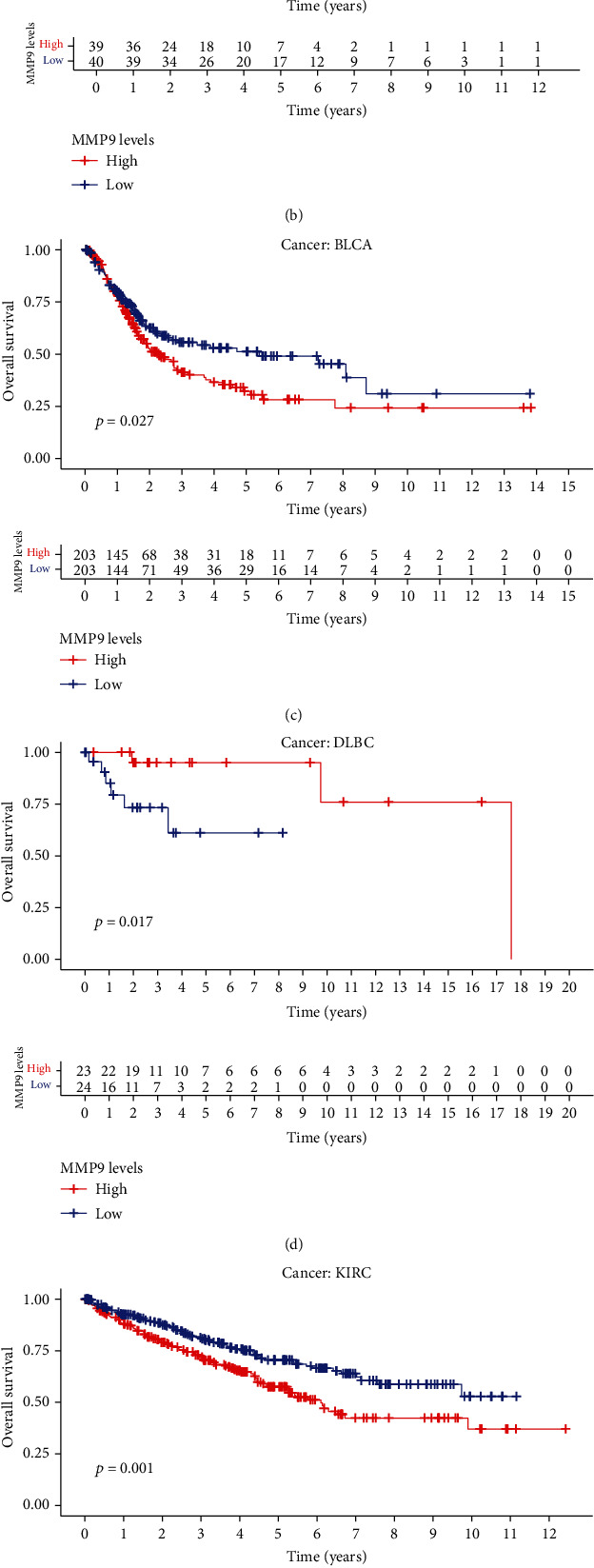
Correlation between MMP-9 and overall survival for various cancer types of TCGA database. (a) Multivariate Cox regression analysis to identify prognosis in 33 cancer types. (b–e) Kaplan-Meier survival curves comparing the high and low expression levels of MMP-9 in different types of cancer. The high expression of MMP-9 was related to the low overall survival rate (b) in ACC (*P* = 0.003), (c) in BLCA (*P* = 0.027), (d) in KIRC (*P* = 0.001), and (e) in LIHC (*P* = 0.009). The low expression of MMP-9 was related to the low overall survival rate of (f) in DLBC (*P* = 0.017).

Next, we investigated whether the expression level of MMP-9 is associated with patient prognosis. Using univariate survival analysis, we found a significant correlation between prognosis and MMP-9 expression in many cancer types, including uterine, kidney, skin, brain, liver, and bladder cancers. Additionally, we used the Kaplan-Meier method to plot the survival curves and found that ACC (*P* = 0.003), BLCA (*P* = 0.027), KIRC (*P* = 0.001), and LIHC (*P* = 0.009) patients with high MMP-9 levels had a poor prognosis (Figures 3(b)–3(e)). However, DLBC patients with high MMP-9 expression had a better prognosis (*P* = 0.017) (Figure 3(f)).

**Figure 4:**
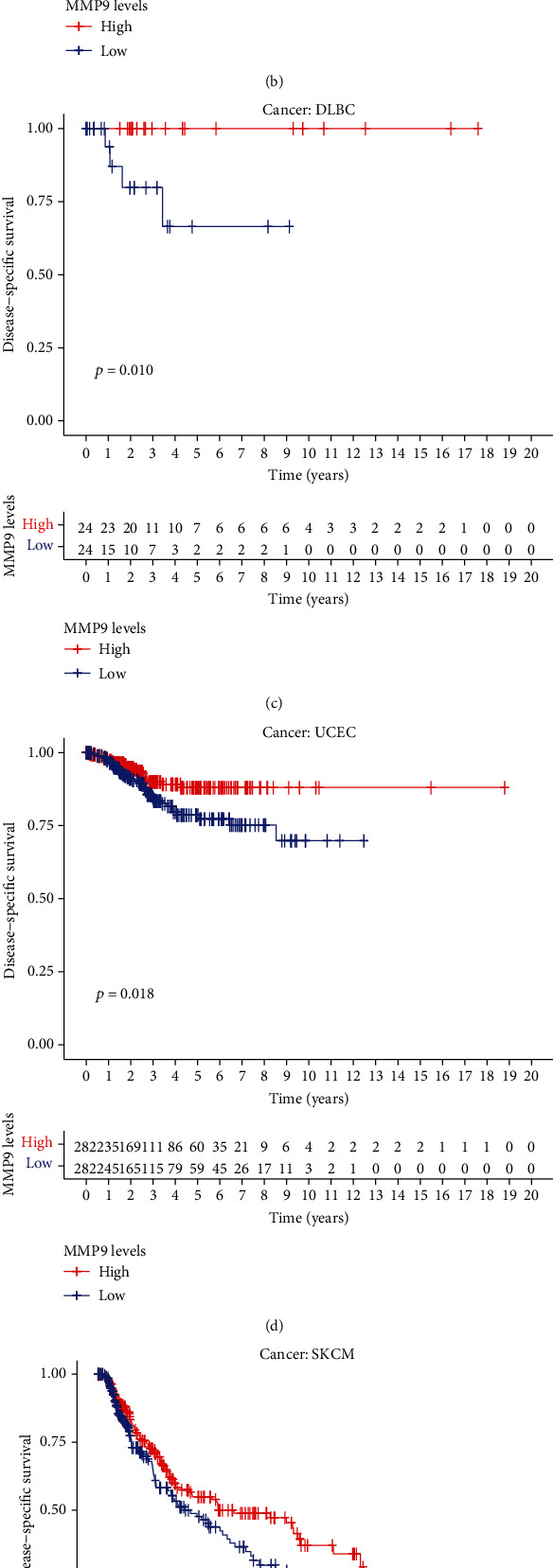
Correlation between MMP-9 and DSS for various cancer types of TCGA database. (a–e) Kaplan-Meier survival curves comparing the high and low expression levels of MMP-9 in different types of cancer. The high expression of MMP-9 was related to the low DSS (a) in ACC (*P* = 0.003) and (b) in KIRC (*P* = 0.018). The low expression of MMP-9 was related to the low DSS (c) in DLBC (*P* = 0.010), (d) in UCEC (*P* = 0.018), and (e) in SKCM (*P* = 0.02) and (f) multivariate Cox regression analysis to identify prognosis in 33 cancer types.

Considering the possibility that there may also be non-tumor-related factors leading to death during the follow-up period, we analyzed the relationship between gene expression and DSS. Notably, MMP-9 expression significantly affected the prognosis in five cancer types (Figures 4(a)–4(e)), including ACC (*P* = 0.003), KIRC (*P* = 0.002), DLBC (*P* = 0.010), UCEC (*P* = 0.018), and SKCM (*P* = 0.029). These results suggest that high MMP-9 expression is an independent risk factor for poor prognosis in ACC and KIRC.

**Figure 5:**
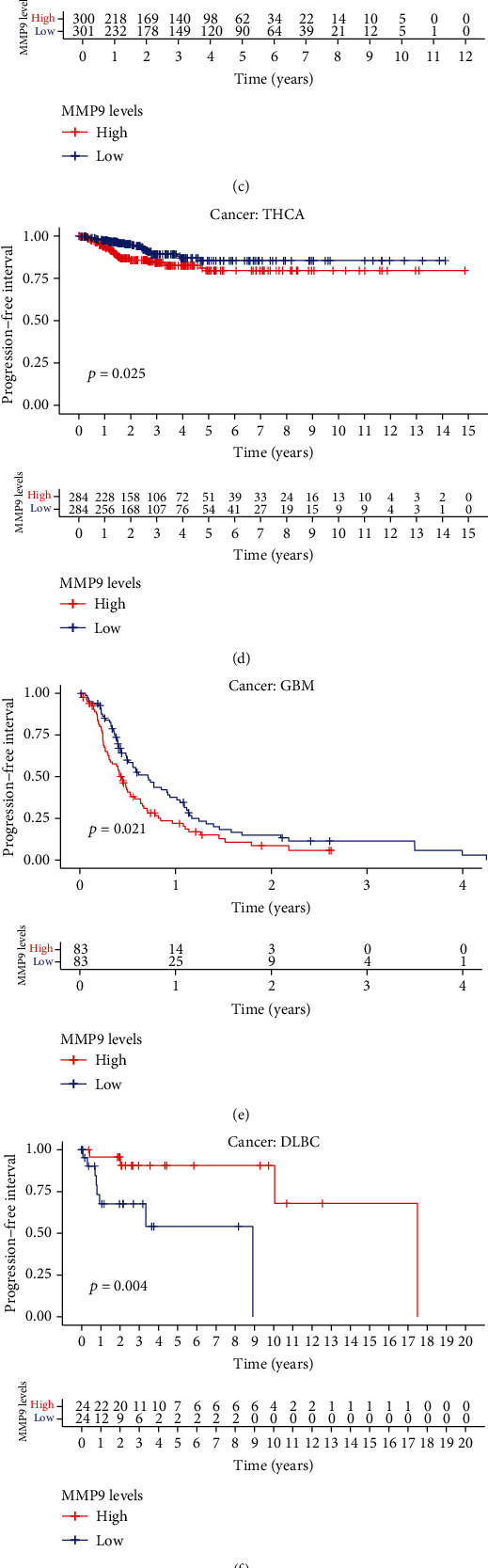
Correlation between MMP-9 and PFI for various cancer types of TCGA database. (a–g) Kaplan-Meier survival curves comparing the high and low expression levels of MMP-9 in different types of cancer. The high expression of MMP-9 was related to the low PFI (a) in ACC (*P* = 0.002), (b) in UVM (*P* = 0.009), (c) in KIRC (*P* = 0.001), (d) in THCA (*P* = 0.025), and (e) in GBM (*P* = 0.021). The low expression of MMP-9 was related to the low PFI (f) in DLBC (*P* = 0.004) and (g) in CESC (*P* = 0.031) and (h) multivariate Cox regression analysis to identify prognosis in 33 cancer types.

To further examine the prognostic potential of MMP-9 in different cancers, we evaluated the PFI of the 33 cancer types. Higher MMP-9 expression levels were associated with shorter PFI in ACC (*P* = 0.002), uveal melanoma (UVM) (*P* = 0.009), KIRC (*P* = 0.001), thyroid carcinoma (THCA) (*P* = 0.025), and GBM (*P* = 0.021) and longer PFI in DLBC (*P* = 0.004) and CESC (*P* = 0.031) (Figures 5(a)–5(g)).

These results indicate that high MMP-9 expression might be a risk factor for poor prognosis in ACC, BLCA, KIRC, LIHC, UVM, THCA, and GBM, while low MMP-9 expression might be a risk factor for poor prognosis in DLBC, UCEC, SKCM, and CESC.

### 3.3. Relationship between MMP-9 Expression and the Clinical Stage

**Figure 6:**
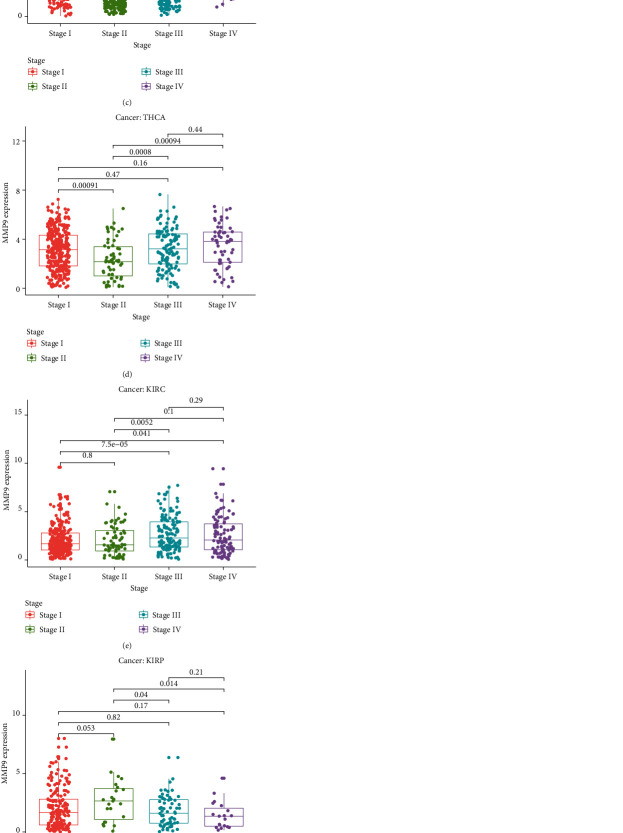
MMP-9 expression in different tumor stages of TCGA database (a) in ACC, (b) in BLCA, (c) in BRCA, (d) in THCA, (e) in KIRC, (f) in KIRP, (g) in SKCM, and (h) in ESCA.

Next, we analyzed the expression of MMP-9 in relation to the tumor stage in the 33 cancer types and found that it was closely related to the clinical stage in eight tumors (Figures 6(a)–6(h)). MMP-9 was differentially expresses according to the clinical stage and was specifically positively correlated with the tumor stage in ACC, BLCA, and KIRC, in which MMP-9 expression increased with tumor progression. These results suggest that MMP-9 expression has the potential to influence cancer prognosis by affecting lymph node metastasis. These results suggest that MMP-9 is involved in promoting cancer progression or metastasis.

### 3.4. Correlation between MMP-9 Expression and Immune Cell Infiltration

**Figure 7:**
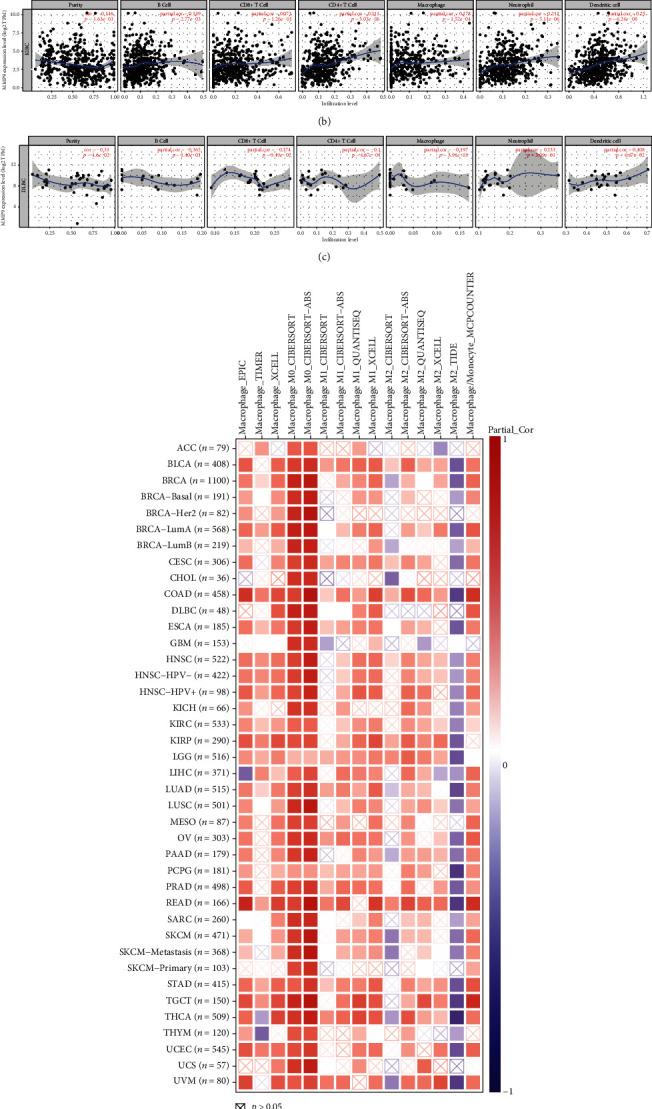
(a–c) Correlation analysis between MMP-9 expression and six kinds of infiltrating immune cells by TIMER database (a) in ACC, (b) in KIRC, and (c) in DLBC and (d) correlation analysis between MMP-9 expression and immune infiltration of macrophage by TIMER 2.0 database.

**Figure 8:**
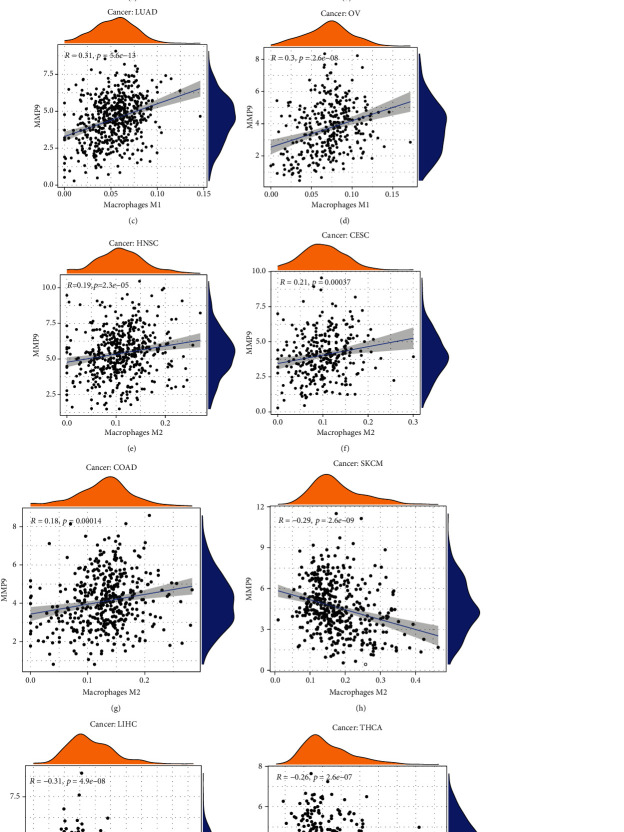
Correlation between MMP-9 gene expression and infiltrating levels of macrophage M1 and macrophage M2 of TCGA database in pan-cancer. MMP-9 was positively correlated with macrophage M1 (a) in CESC, (b) in LGG, (c) in LUAD, and (d) in OV. MMP-9 was positively correlated with macrophage M2 (e) in HNSC, (f) in CESC, and (g) in COAD. MMP-9 was negatively correlated with macrophage M2 (h) in SKCM, (i) in LIHC, and (j) in THCA.

Many studies have shown that MMP-9 is related to immune cells [33, 34]. Therefore, we evaluated the correlation between MMP-9 and immune cell infiltration in 33 tumors. Through survival analysis and clinical correlation analysis, we found that MMP-9 was related to poor prognosis and metastasis in ACC and KIRC. DLBC was used as the control group. The correlation between the expression level of MMP-9 and six types of infiltrating immune cells in ACC, KIRC, and DLBC is shown in Figures 7(a)–7(c). The expression of MMP-9 was positively correlated with the infiltration of B cells, CD8+ cells, CD4+ cells, and macrophages in ACC and KIRC, while it was mostly negatively correlated in DLBC. In addition, our results indicated a marked correlation between MMP-9 expression and the macrophage M0 in 28 cancer types (Table 1). MMP-9 was positively correlated with the macrophage M1 in four tumors (Figures 8(a)–8(d)). The levels of infiltrating macrophage M2 were positively correlated with MMP-9 expression in HNSC, CESC, and COAD (Figures 8(e)–8(g)) and negatively correlated in SKCM, LIHC, and THCA (Figures 8(h)–8(j)). In addition, TIMER2.0 analysis showed that MMP-9 had a strong positive correlation with macrophages (Figure 7(d)). These results showed that high MMP-9 expression was positively correlated with immune cell infiltration.

### 3.5. Correlation between the MMP-9 Expression Level and Immune Cell Markers

The TME [35] can affect survival and tumor metastasis. We performed immune cell marker gene coexpression analyses in ACC, KIRC, and DLBC and found that the expression of MMP-9 was mainly positively correlated with the expression levels of most marker sets of T cells, TAMs, M2 macrophages, Th1 cells, and T cell exhaustion, especially in ACC (Table 2), while no such correlation was observed in DLBC.

### 3.6. Coexpression of DNA Repair Genes with MMP-9 and GSEA

**Figure 9:**
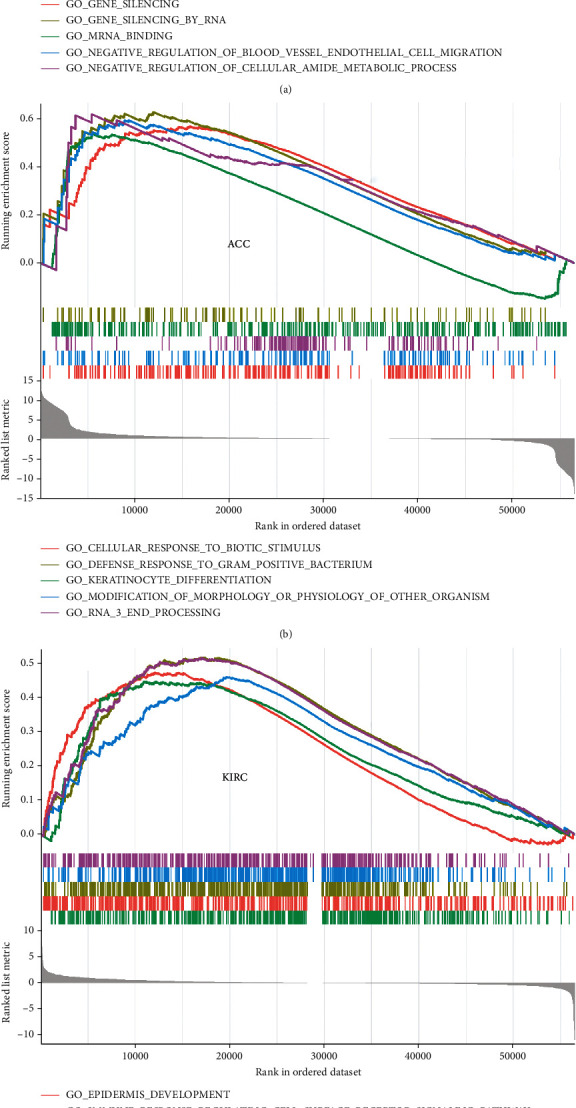
Pathway analysis of MMP-9 in different cancers and DNA repair gene coexpression analysis with MMP-9 of TCGA database. (a) GO functional annotation of MMP-9 in DLBC, (b) GO functional annotation of MMP-9 in ACC, (c) GO functional annotation of MMP-9 in KIRC, and (d) DNA repair gene coexpression analysis with MMP-9. Each small rectangular module represents the coexpression of DNA repair genes and MMP-9 in cancer, where the upper left corner is the *P* value, where ^∗^*P* < 0.05, ^∗∗^*P* < 0.01, and ^∗∗∗^*P* < 0.001, and the lower right corner is the correlation coefficient.

To better understand the potential mechanism of MMP-9 expression in cancers, we analyzed its expression in ACC, KIRC, and DLBC using GSEA. The results showed that MMP-9 was mainly enriched in immune-related pathways in KIRC, such as immune response regulating cell surface receptor signaling and regulation of immune effector process (Figure 9(c)), and in pathways related to gene silencing and RNA modification in ACC and DLBC (Figures 9(a) and 9(b)). We further used RNA sequence data from TCGA database to evaluate the correlation between MMP-9 and five DNA repair genes and found that MMP-9 was associated with multiple DNA repair genes in various tumors (Figure 9(d)). More specifically, MMP-9 was moderately positively correlated with MSH2 in ACC and negatively correlated with EPCAM and PMS2 in KIRC. In addition, MMP-9 showed a significant correlation with DNA repair genes in LGG and LIHC.

### 3.7. Correlation between the MMP-9 Expression Level and TMB, MSI, and Immune Checkpoint Genes

**Figure 10:**
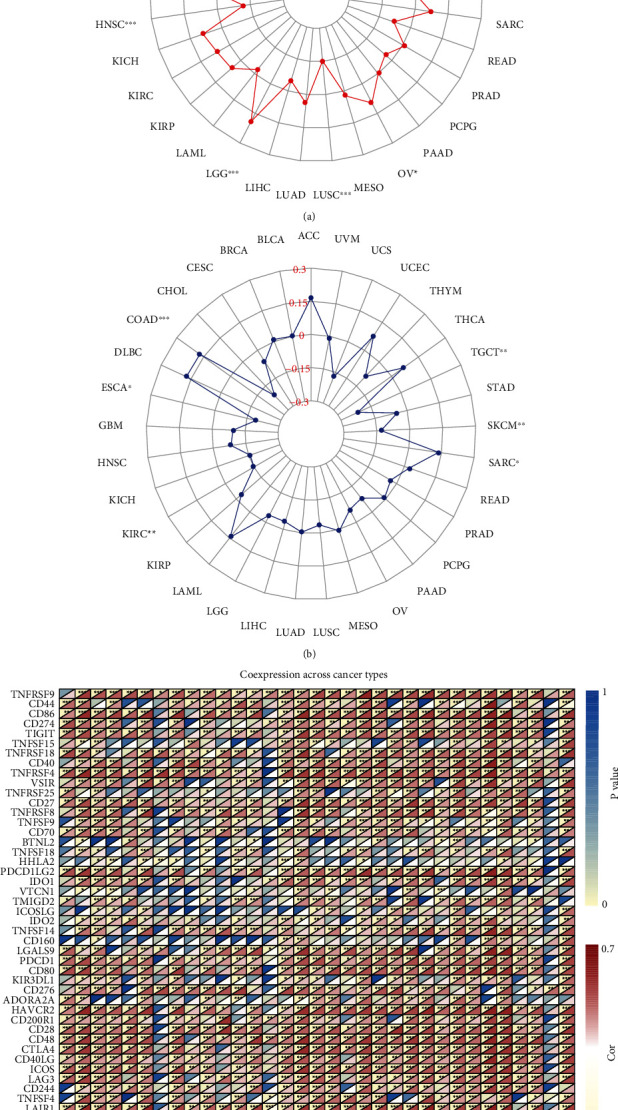
Correlation between MMP-9 gene expression and TMB and MSI and coexpression between MMP-9 and immunological checkpoint genes of TCGA database in pan-cancer. (a) Correlation between MMP-9 and TMB in 33 cancer types. (b) Correlation between MMP-9 and MSI in 33 cancer types. (c) Coexpression of MMP-9 and immunological checkpoint genes, ^∗^*P* < 0.05, ^∗∗^*P* < 0.01, and ^∗∗∗^*P* < 0.001.

TMB and MSI are important for immunotherapy response. Here, we calculated the TMB of each tumor sample and analyzed the correlation between MMP-9 and TMB in 33 tumors. MMP-9 was positively correlated with TMB in six tumors, including ACC, BRCA, COAD, brain lower grade glioma (LGG), OV, and UCEC, and negatively correlated with HNSC and LUSC (Figure 10(a)). Next, we analyzed the correlation between MSI and MMP-9 levels. MSI was positively correlated with MMP-9 in COAD and sarcoma, whereas it was negatively correlated in four tumors (Figure 10(b)). In addition, most immune checkpoint genes were coexpressed with MMP-9, especially PDCD1 and CTL4, which are the targets of immune checkpoint inhibitors.

## 4. Discussion

MMP-9 can degrade the extracellular matrix components and promote tumor invasion and metastasis. The high expression of MMP-9 is closely related to the development, invasion, and metastasis in many cancers. Here, we found that MMP-9 promotes cancer development and progression in some cancers, suggesting that MMP-9 expression can be used to predict metastasis, especially in kidney cancer. In addition, correlation analysis showed that the expression of MMP-9 was correlated with different levels of immune infiltration and immunological markers. Finally, we evaluated the relationship between MMP-9 expression and TMB and MSI. The results showed that MMP-9 may be used as a biomarker for pan-cancer prognosis.

In this study, we obtained the expression levels of MMP-9 and the prognosis and relevant indices of 33 cancer types from TCGA database. Differential expression of MMP-9 in cancer and normal tissues was observed in all cancers, with MMP-9 being overexpressed in tumor tissue across cancer types. This suggested that dysregulated or excessive MMP-9 could cause tumorigenesis. As for the survival analysis, higher expression levels of MMP-9 were correlated with poorer prognosis in patients with ACC, BLCA, KIRC, and LIHC. In contrast, high levels of MMP-9 were favorable for the prognosis of lymphoma. The results indicated that MMP-9 promotes bladder and cervical cancer invasion and metastasis. MMP-9 is a potential prognostic biomarker for various cancers, including lung, ovarian, pancreatic, and breast cancers [11, 16]. However, in our study, analysis based on three survival indicators showed that high MMP-9 expression was associated with poor prognosis in ACC and KIRC. The correlation between MMP-9 and renal cancers has not been reported in previous studies. In addition, our analysis of OS, DSS, and PFI showed that high expression of MMP-9 is a protective factor in DLBC; however, this has not been observed in previous studies. MMP-9 promotes metastasis via ECM decomposition [36]. The expression of MMP-9 was related to the clinical stage in eight tumors, suggesting that MMP-9 may be involved in tumor metastasis. In addition, MMP-9 increased with the progression of cancer in three types of urological tumors. These results suggest that MMP-9 may be used as an indicator of prognosis and metastasis in pan-cancer.

Furthermore, we found that MMP-9 expression was correlated with immune infiltration levels in multiple cancer types, especially ACC and KIRC. It was positively correlated with the infiltration of B cells, CD8+ cells, CD4+ cells, and macrophages in ACC and KIRC, while it was mostly negatively correlated in DLBC. This suggests that MMP-9 may lead to poor prognosis by participating in tumor immune infiltration. Moreover, MMP-9 expression levels were mainly positively correlated with immune cell markers. Notably, in ACC, MMP-9 was moderately correlated with four Th1 marker genes (TBX21, STAT4, STAT1, and IFNG), suggesting that it may be involved in Th1 differentiation. Th1 cells induce the activation of macrophages, NK cells, B cells, and CD8+ T cells [37]. Concurrently, we also found that MMP-9 was moderately correlated with the immune markers of CD8+ T cells (CD8A and CD8B) and T cells (CD3D, CD3E, and CD2). These results suggest that MMP-9 may promote cell-mediated inflammatory responses by participating in Th1 differentiation and T cell activation. Th1 cells regulate macrophage function at multiple levels. In addition, MMP-9 was associated with macrophage immune marker genes. More specifically, MMP-9 expression was positively correlated with IL-10 (a TAM marker), which is often associated with tumor immune evasion. The markers of M2 macrophages were moderately correlated with MMP-9 expression in tumors, suggesting that MMP-9 may be involved in the differentiation of macrophages. Most importantly, in ACC, MMP-9 expression was strongly correlated with most markers of T cell exhaustion, including TGFB1, PDCD1, CTLA4, LAG3, and GZMB. T cell exhaustion is one of the main causes of immune dysfunction that leads to a poor prognosis [38]. This suggests that MMP-9 may be the cause of poor prognosis in patients with ACC. At the same time, T cell exhaustion is also one of the reasons for poor immunotherapy response. In contrast, TAMs are important cellular components of the TME [39] and imbalance of M1/M2 plays a key role in tumor progression, immune escape, and drug resistance [40]. Therefore, the development of antineoplastic drugs that target macrophage polarization is important. Tekin et al. [18] found that M0 macrophages secrete MMP-9 in the early stages of pancreatic cancer development, which promotes tumor progression. This is consistent with the findings of our study. In addition, we found that MMP-9 was highly positively correlated with M0 macrophage levels in 27 types of tumors. Although research has shown that M2 macrophages can alter miR-149-5p to increase the expression of MMP-9 in liver cancer [41], in our study, MMP-9 and M2 macrophages were negatively correlated in LIHC. These results indicated that MMP-9 is involved in the recruitment and activation of immune cells and that MMP-9 inhibition may be another approach for tumor immunotherapy based on macrophages.

In this study, MMP-9 expression was associated with TMB in eight cancer types and with MSI in six cancer types. In ACC, MMP-9 was highly correlated with the markers of T cell exhaustion, which can be reversed by PD-1 inhibitors. A recent study [42] identified TMB as a marker for evaluating the therapeutic effect of PD-1 inhibitors. Therefore, we analyzed the relationship between MMP-9 expression and TMB expression. Our results also showed that MMP-9 has a significant positive correlation with TMB in a variety of cancers. This suggests that in these cancers, patients with high MMP-9 expression may be more suitable for immunosuppressive therapy. Furthermore, MSI plays an important role in the diagnosis, prognosis, and treatment of multiple tumors, especially colon cancer [43]. Our results showed that MSI is positively correlated with MMP-9 in COAD. In brief, patients with high MMP-9 expression may be more suitable for immunotherapy.

Immune checkpoints are closely related to tumor immune escape. Hence, we analyzed the relationship between the expression of MMP-9 and certain common immune checkpoint genes. The results showed that MMP-9 was significantly associated with immune checkpoints in most tumor types. This may be related to the poor prognosis of some tumors in the survival analysis. Another study [44] indicated that inhibition of MMP-2/MMP-9 improves the efficacy of PD-1 or CTLA4 blockade in the treatment of primary and metastatic tumors.

Monferran et al. [45] reported that the DNA repair protein Ku interacts with MMP-9 at the cell membrane of highly invasive hematopoietic cells. Our results also showed that MMP-9 was correlated with various DNA repair genes. These findings may help in understanding the role of MMP-9 in gene expression and gene repair. The GSEA results also suggested that MMP-9 participates in immune regulation. This is consistent with the results of our previous analysis. This suggests that MMP-9 is a potential target for immunotherapy.

Although we comprehensively analyzed MMP-9 expression in 33 tumors, many deficiencies exist in our study. First, our data source was relatively single and simple as we used mainly TCGA database data. Second, our findings require further validation in the clinical setting. Third, although we found that the expression of MMP-9 is related to immune cell infiltration and survival, we could not prove its causal relationship, and hence, its prognostic value needs to be further studied.

In conclusion, MMP-9 can be used as a pan-cancer prognostic biomarker involving immune infiltration, especially in kidney cancer. These findings may contribute to clinical decision-making and cancer immunotherapy.

## Figures and Tables

**Table 1:** Correlation analysis between MMP-9 and macrophage M0 of TCGA database (the *P* values are indicated as ^∗^*P* < 0.05, ^∗∗^*P* < 0.01, and ^∗∗∗^*P* < 0.001).

Cancer type	Cor	*P* value
ACC	0.59	^∗∗∗^
BLCA	0.43	^∗∗∗^
BRCA	0.58	^∗∗∗^
CESC	0.29	^∗∗∗^
COAD	0.36	^∗∗∗^
DLBC	0.56	^∗∗∗^
ESCA	0.29	^∗∗∗^
GBM	0.61	^∗∗∗^
HNSC	0.32	^∗∗∗^
KICH	0.67	^∗∗∗^
KIRC	0.52	^∗∗∗^
KIRP	0.61	^∗∗∗^
LGG	0.49	^∗∗∗^
LIHC	0.24	^∗∗∗^
LUAD	0.23	^∗∗∗^
LUSC	0.29	^∗∗∗^
MESO	0.42	^∗∗∗^
PAAD	0.32	^∗∗∗^
PCPG	0.61	^∗∗∗^
PRAD	0.62	^∗∗∗^
SARC	0.64	^∗∗∗^
SKCM	0.30	^∗∗∗^
STAD	0.26	^∗∗∗^
TGCT	0.46	^∗∗∗^
THCA	0.23	^∗∗∗^
THYM	0.33	^∗∗∗^
UCEC	0.29	^∗∗∗^
UCS	0.72	^∗∗∗^

**Table 2:** Correlation analysis between MMP-9 and related genes and markers of immune cells of TCGA database (the *P* values are indicated as ^∗^*P* < 0.05, ^∗∗^*P* < 0.01, and ^∗∗∗^*P* < 0.001).

Description	Gene markers	ACC		KIRC		DLBC	
		Cor	*P* value	Cor	*P* value	Cor	*P* value
CD8+ T cell	CD8A	0.336	^∗∗^	0.111	^∗^	0.098	0.506
	CD8B	0.363	^∗∗∗^	0.095	^∗^	0.021	0.886

T cell (general)	CD3D	0.405	^∗∗∗^	0.192	^∗∗∗^	0.130	0.379
	CD3E	0.371	^∗∗∗^	0.201	^∗∗∗^	0.098	0.510
	CD2	0.342	^∗∗^	0.182	^∗∗∗^	0.123	0.403

B cell	CD19	−0.040	0.723	0.306	^∗∗∗^	0.081	0.583
	CD79A	0.050	0.664	0.316	^∗∗∗^	0.066	0.654

Monocyte	CD86	0.292	^∗∗^	0.248	^∗∗∗^	0.101	0.493
	CSF1R	0.220	0.051	0.255	^∗∗∗^	0.231	0.114

TAM	CCL2	0.071	0.537	−0.071	0.102	0.237	0.105
	CD68	0.254	0.024	0.255	^∗∗∗^	0.343	0.017
	IL-10	0.545	^∗∗∗^	0.302	^∗∗∗^	0.360	0.012

M1 macrophage	NOS2	0.408	^∗∗∗^	−0.063	0.148	0.176	0.233
	IRF5	0.169	0.137	0.062	0.153	0.049	0.739
	PTGS2	0.505	^∗∗∗^	0.228	^∗∗∗^	0.151	0.305

M2 macrophage	CD163	0.400	^∗∗∗^	0.305	^∗∗∗^	0.176	0.232
	VSIG4	0.350	^∗∗^	0.342	^∗∗∗^	0.120	0.418
	MS4A4A	0.375	^∗∗∗^	0.302	^∗∗∗^	0.340	0.018

Neutrophils	CEACAM8 (CD66b)	0.187	0.098	0.004	0.925	0.150	0.310
	ITGAM (CD11b)	0.290	^∗∗^	0.202	^∗∗∗^	0.487	0.000

Natural killer cell	KIR2DL1	0.101	0.376	−0.029	0.503	0.047	0.753
	KIR2DL3	0.034	0.765	−0.060	0.166	0.063	0.671
	KIR2DL4	0.318	^∗∗^	0.085	^∗^	0.106	0.473
	KIR3DL1	0.142	0.211	−0.111	^∗^	0.141	0.338
	KIR3DL2	−0.202	0.074	−0.014	0.754	0.103	0.485
	KIR3DL3	0.159	0.161	0.026	0.541	0.043	0.771
	KIR2DS4	0.145	0.203	0.014	0.755	0.065	0.658

Dendritic cell	HLA-DPB1	0.161	0.157	0.147	^∗∗∗^	0.196	0.182
	HLA-DQB1	0.117	0.306	0.037	0.391	0.128	0.384
	HLA-DRA	0.137	0.227	0.143	^∗∗∗^	0.120	0.418
	HLA-DPA1	0.075	0.510	0.118	^∗∗^	0.131	0.376
	NRP1 (BDCA-4)	0.239	0.034	0.044	0.305	0.139	0.348
	CD1C (BDCA-1)	0.011	0.924	0.090	^∗^	0.009	0.952
	ITGAX (CD11c)	0.340	^∗∗^	0.271	^∗∗∗^	0.536	0.000

Th1	TBX21	0.434	^∗∗∗^	0.053	0.224	0.076	0.610
	STAT4	0.463	^∗∗∗^	0.178	^∗∗∗^	0.067	0.653
	STAT1	0.301	^∗∗^	0.062	0.154	0.096	0.517
	IFNG (TNF-*γ*)	0.397	^∗∗∗^	0.098	^∗^	0.137	0.354
	TNF (TNF-*α*)	0.070	0.541	0.069	0.110	0.204	0.165

Th2	GATA3	0.026	0.820	0.044	0.308	0.134	0.363
	STAT6	−0.206	0.069	−0.054	0.216	0.365	0.011
	STAT5A	0.198	0.080	0.193	^∗∗∗^	0.117	0.429
	IL-13	0.035	0.762	0.044	0.311	0.099	0.504

Tfh	BCL6	0.087	0.445	0.191	^∗∗∗^	0.100	0.498
	IL-21	0.000	1.000	0.165	^∗∗∗^	0.061	0.680

Th17	STAT3	0.200	0.078	0.087	^∗^	0.371	0.009
	IL-17A	0.000	1.000	0.065	0.135	0.023	0.879

Treg	FOXP3	0.162	0.153	0.385	^∗∗∗^	0.196	0.181
	CCR8	0.015	0.899	0.250	^∗∗∗^	0.181	0.217
	STAT5B	0.017	0.880	−0.193	^∗∗∗^	0.229	0.118

T cell exhaustion	TGFB1	0.522	^∗∗∗^	0.411	^∗∗∗^	0.090	0.542
	PDCD1 (PD-1)	0.399	^∗∗∗^	0.141	^∗∗^	0.007	0.961
	CTLA4	0.392	^∗∗∗^	0.155	^∗∗∗^	0.177	0.230
	LAG3	0.412	^∗∗∗^	0.169	^∗∗∗^	0.026	0.862
	HAVCR2 (TAM-3)	0.299	^∗∗^	0.040	0.358	0.054	0.715
	GZMB	0.551	^∗∗∗^	0.140	^∗∗^	0.163	0.269

## Data Availability

The datasets obtained from UCSC Xena (http://xena.ucsc.edu/), partial analysis by GEPIA2 database (http://gepia2.cancer-pku.cn/#analysis) TIMER (https://cistrome.shinyapps.io/timer/), and TIMER2.0 database (http://timer.comp-genomics.org/).

## Abbreviations

MMP-9: Matrix metalloproteinase-9
TMB: Tumor mutation burden
DSS: Disease-specific survival
PFI: Progression-free interval
TME: The tumor microenvironment
MSI: DNA microsatellite instability
ACC: Adrenocortical carcinoma
BLCA: Bladder urothelial carcinoma
BRCA: Breast invasive carcinoma
CESC: Cervical squamous cell carcinoma and endocervical adenocarcinoma
CHOL: Cholangiocarcinoma
COAD: Colon adenocarcinoma
DLBC: Lymphoid neoplasm diffuse large B cell lymphoma
ESCA: Esophageal carcinoma
GBM: Glioblastoma multiforme
HNSC: Head and neck squamous cell carcinoma
KICH: Kidney chromophobe
KIRC: Kidney renal clear cell carcinoma
KIRP: Kidney renal papillary cell carcinoma
LAML: Acute myeloid leukemia
LGG: Brain lower grade glioma
LIHC: Liver hepatocellular carcinoma
LUAD: Lung adenocarcinoma
LUSC: Lung squamous cell carcinoma
MESO: Mesothelioma
OV: Ovarian serous cystadenocarcinoma
PAAD: Pancreatic adenocarcinoma
PCPG: Pheochromocytoma and paraganglioma
PRAD: Prostate adenocarcinoma
READ: Rectum adenocarcinoma
SARC: Sarcoma
SKCM: Skin cutaneous melanoma
STAD: Stomach adenocarcinoma
TGCT: Testicular germ cell tumors
THCA: Thyroid carcinoma
THYM: Thymoma
UCEC: Uterine corpus endometrial carcinoma
UCS: Uterine carcinosarcoma
UVM: Uveal melanoma.
